# MicroRNA–mRNA networks are dysregulated in opioid use disorder postmortem brain: Further evidence for opioid-induced neurovascular alterations

**DOI:** 10.3389/fpsyt.2022.1025346

**Published:** 2023-01-12

**Authors:** Sandra L. Grimm, Emily F. Mendez, Laura Stertz, Thomas D. Meyer, Gabriel R. Fries, Tanmay Gandhi, Rupa Kanchi, Sudhakar Selvaraj, Antonio L. Teixeira, Thomas R. Kosten, Preethi Gunaratne, Cristian Coarfa, Consuelo Walss-Bass

**Affiliations:** ^1^Dan L Duncan Comprehensive Cancer Center, Baylor College of Medicine, Houston, TX, United States; ^2^Department of Molecular and Cellular Biology, Baylor College of Medicine, Houston, TX, United States; ^3^Center for Precision Environmental Health, Baylor College of Medicine, Houston, TX, United States; ^4^Louis A. Faillace, MD, Department of Psychiatry and Behavioral Sciences, McGovern Medical School, University of Texas Health Science Center at Houston, Houston, TX, United States; ^5^Department of Neuroscience, Baylor College of Medicine, Houston, TX, United States; ^6^Department of Psychiatry, Baylor College of Medicine, Houston, TX, United States; ^7^Department of Biology and Biochemistry, University of Houston, TX, United States

**Keywords:** opioid use disorder, microRNA, brain, network analysis, neurovascular, blood, prefrontal cortex

## Abstract

**Introduction:**

To understand mechanisms and identify potential targets for intervention in the current crisis of opioid use disorder (OUD), postmortem brains represent an under-utilized resource. To refine previously reported gene signatures of neurobiological alterations in OUD from the dorsolateral prefrontal cortex (Brodmann Area 9, BA9), we explored the role of microRNAs (miRNA) as powerful epigenetic regulators of gene function.

**Methods:**

Building on the growing appreciation that miRNAs can cross the blood-brain barrier, we carried out miRNA profiling in same-subject postmortem samples from BA9 and blood tissues.

**Results:**

miRNA–mRNA network analysis showed that even though miRNAs identified in BA9 and blood were fairly distinct, their target genes and corresponding enriched pathways overlapped strongly. Among the dominant enriched biological processes were tissue development and morphogenesis, and MAPK signaling pathways. These findings point to robust, redundant, and systemic opioid-induced miRNA dysregulation with a potential functional impact on transcriptomic changes. Further, using correlation network analysis, we identified cell-type specific miRNA targets, specifically in astrocytes, neurons, and endothelial cells, associated with OUD transcriptomic dysregulation. Finally, leveraging a collection of control brain transcriptomes from the Genotype-Tissue Expression (GTEx) project, we identified a correlation of OUD miRNA targets with TGF beta, hypoxia, angiogenesis, coagulation, immune system, and inflammatory pathways.

**Discussion:**

These findings support previous reports of neurovascular and immune system alterations as a consequence of opioid abuse and shed new light on miRNA network regulators of cellular response to opioid drugs.

## 1. Introduction

Opioid use disorder (OUD) is a major public health problem responsible for alarming rates of overdoses and deaths, yet the neurobiological consequences of long-term opioid misuse are not well understood. Opioid use is known to cause gene function dysregulation at multiple levels, including DNA histone modification (1, 2), DNA methylation (2–4), protein expression (5, 6), mRNA and long-non-coding RNA expression (5, 7), and single-cell gene expression (8). Opioid drugs also affect cellular microRNAs (miRNAs), 17–22 nucleotide sequences that act as pleiotropic regulators of mRNAs through imperfect matching, mostly at the 3′ UTR of genes (9–11). miRNAs are key modulators of intercellular communication in the brain (12) across multiple species (13) and are dysregulated in psychiatric disorders such as schizophrenia, bipolar disorder, and substance use disorder (14). Opioid exposure significantly alters miRNA expression in many regions of the brain, but most notably in the prefrontal cortex, striatum, and nucleus accumbens (15, 16). Several miRNAs involved in regulation of synaptic plasticity are hypothesized to underlie drug addiction (17) and miRNAs have been shown to regulate μ-opioid receptor levels and modulate opioid tolerance (18, 19). These studies point toward miRNAs as critical epigenetic modulators of short- and long-term opioid effects in the brain through regulation of gene expression.

Importantly, miRNAs can cross the blood-brain barrier (20, 21), potentially via exosomes (22–24), and assessment of differential miRNA expression in blood has been used to identify surrogate blood-based biomarkers in brain diseases, including Alzheimer’s disease, depression, and cancer (25–27). Brain-specific miRNAs identified in peripheral blood have been proposed as markers for traumatic brain injury (28). Additionally, a recent study found differential expression of miRNAs in heroin- and methamphetamine-dependent patients that functionally predicted anxiety and depression symptoms (29).

Bioinformatics pipelines are powerful tools that can extract critical drivers of signaling pathways underlying OUD-related changes in the brain. These pipelines have been developed to identify biosignatures and new druggable targets from miRNA-regulated networks inferred using miRNA and mRNA profiles measured in the same specimens. This approach exceeds both the single gene discovery approach as well as the use of large gene signatures that, due to their diffuse biological impact, might otherwise prove intractable for elucidation of mechanisms and effective repurposing of pharmacotherapeutic agents. No studies have used a comprehensive bioinformatics approach to integrate miRNA expression and their regulated transcripts in postmortem tissues from subjects with OUD. Integrating these networks in brain and blood from the same subjects will identify functional biomarkers of dysregulation of target genes in brain regions affected by OUD and unveil a powerful resource for not only validating brain and surrogate blood-based biomarkers for this disease but also for identifying larger-scale gene networks that point to disease pathophysiology in general.

## 2. Materials and methods

### 2.1. Sample information

Postmortem brain and peripheral blood tissues were obtained from the University of Texas Health Science Center at Houston (UTHealth) Brain Collection in collaboration with the Harris County Institute of Forensic Science, with consent from the next of kin (NOK) and approval from the Institutional Review Board. Medical Examiner Reports, including toxicology, were obtained and medical records were acquired when available. The detailed UT Health Psychological Autopsy Interview Schedule was performed on the donor by interviewing the NOK (30) from which information of psychiatric clinical phenotypes (evidence of depression, mania, and psychosis), age of onset of drug use, types of drugs used, smoking and drinking history, and any co-morbidities was obtained. A diagnosis of OUD, or designation as non-psychiatric control (absence of any apparent psychopathology), was determined according to DSM-5 criteria after a consensus meeting where three trained clinicians reviewed the psychological autopsy and all other available records.

Upon receipt of the brain, the right hemisphere was coronally sectioned, immediately frozen, and stored at −80°C. Dissections of BA9, defined within the DLPFC between the superior frontal gyrus and the cingulate sulcus, were obtained using a 4 mm cortical punch. RNA was extracted from 50 mg of tissue using the RNeasy Plus Mini Kit (Qiagen, Hilden, Germany) and RNA integrity number (RIN) was measured for RNA quality (Agilent Bioanalyzer 2100 system, Agilent Technologies, Santa Clara, CA, USA). Postmortem interval (PMI) was calculated from the estimated time of death until tissue preservation. Peripheral blood samples were collected into EDTA-containing tubes, and then stored as whole blood at −80°C until further use.

### 2.2. RNA-seq analysis

We analyzed RNA-seq data generated as previously described (5) for 15 controls and 27 OUD cases using bulk BA9 tissue. RNA-seq data was trimmed for low quality base pairs and adapter sequences using trim_galore. Sequencing reads were mapped to the human genome build UCSC hg38 using STAR (31). Gene expression was quantified using featureCounts (32). The following demographic variables were accounted and regressed out using the R package RUVr (33): Sex, Age, PMI (hours), pH, and RNA integrity number (RIN). We used the R package EdgeR (34) to infer differentially expressed genes between groups, with significance achieved at a fold change exceeding 1.5x and FDR-adjusted *p*-value < 0.05.

### 2.3. smallRNA-seq analysis

The small RNA fraction was isolated from bulk BA9 tissue from 15 controls and 24 OUD cases for which brain tissue was available and bulk RNA-seq data was already generated (5), and from blood from a subset of the same individuals from whom blood tissue was available (8 controls and 18 OUD). miRNA libraries were prepared and sequenced at the University of Houston Seq-N-Edit Core per standard protocols. Briefly, RNA was isolated using the miRNAeasy Mini Kit (Qiagen) and total RNA, including miRNA, was bound to a miRNeasy spin column and washed three times before subsequent elution. Quality checks of the extracted RNA were performed using a Qubit Fluorometer (Thermo Fisher, Waltham, MA, USA) and an RNA tape on a TapeStation 4200 (Agilent). miRNA libraries were prepared with the QIAseq miRNA library kit (Qiagen) using 5 μL of the extracted total RNA sample. Libraries were produced by sequentially ligating adapters to the 3′ and 5′ ends of miRNAs. This was followed by reverse transcription into cDNA and subsequent ligation of sample indexes and sequencing adapters. The size selection for libraries was performed using QIAseq beads (Qiagen). Library purity was analyzed using the DNA HS1000 tape on a Tapestation 4200 (Agilent) and quantified with Qubit Fluorometer (Thermo Fisher, Waltham, MA, USA). Small RNA libraries were sequenced on an Illumina Genome Analyzer NextSeq 500. We analyzed 3–4 million reads per sample using our published bioinformatics pipeline (35). We constructed RNA-seq libraries using the Takara SMARTer Universal Low Input RNA Kit (Takara Bio, Kusatsu, Shiga, Japan) designed to handle 2–100 ng of total RNA and retain strand-specific information.

The smallRNA-seq data was trimmed for low quality base pairs and adapter sequences using trim_galore. Sequencing reads were mapped to the human genome build UCSC hg38 using STAR (31) allowing for at most 10 matches. miRNA expression was quantified using featureCounts (32) and the miRBase reference database (10). The following demographic variables were accounted and regressed out using the R package RUVr (33): Sex, Age, PMI (hours), pH, and RIN. We used the R package EdgeR (34) to screen for differentially expressed miRNAs between groups, considering an unadjusted *p*-value < 0.05. We conducted miRNA–mRNA integration using the SigTerms methodology (36), utilizing the miRDB (37) database as a reference for miRNA–mRNA interactions. Specifically, we employed the hypergeometric distribution to assess enrichment of miRNA targets in a gene list, with significance achieved at FDR-adjusted *p*-value < 0.05. miRNA–mRNA networks were visualized using the Cytoscape platform (38). Correlation of specific miRNAs detected both in blood and in BA9 samples with expression measured as log2(CPM) (counts per million reads mapped) was assessed using the Pearson Correlation Coefficient as implemented in the R statistical system; significance was achieved for *p* < 0.05. SmallRNA-seq data will be deposited in the NCBI Gene Expression Omnibus.

### 2.4. Pathway enrichment analysis

Enriched pathways were determined using the Gene Ontology Biological Processes (GOBP) compendium as compiled by the Molecular Signatures Database (MSigDB) (39) based on hypergeometric distribution, with significance achieved at FDR < 0.05 and the requirement to have at least two genes from an input gene list present in a GOBP pathway. To determine GO terms enriched in collections of significant GOBP pathways, we used an approach similar to the one employed by the REVIGO method (40). Specifically, we determined significance of enrichment or depletion of a GO term in a collection of pathways using a Fisher’s exact test, with significance achieved for FDR < 0.05; we further determined enriched GO terms by requiring the odds-ratio to be greater than 1.

### 2.5. Gene network analysis

Gene network analysis was carried out using the Weighted Correlation Network Analysis (WGCNA) R package (40), using default parameters and a minimum module size of 5. Brain cell type composition was inferred with CIBERSORT (41) using reference gene signatures previously reported (42) for human brain cell types and default parameters. Significant association with clinical traits was performed using WGCNA, with significance achieved at *p* < 0.05.

### 2.6. Drug repurposing analysis

Repurposing of drugs targeting the gene signatures determined using the miRNA–mRNA analysis pipeline was performed using the Library of Integrated Network-Based Cellular Signatures (LINCS) Program/Connectivity Map platform at:https://clue.io (43).

### 2.7. Signature correlation analysis

A methodology used frequently to assess the interaction between gene signatures in human cohorts is the correlation of gene signature scores; this approach has been utilized both in cancer (44–46) and non-cancer systems (47–49). We downloaded data for 425 control bulk BA9 brain tissues collected by the Genotype-Tissue Expression (GTEx) project (50), using the GTEx data portal. We computed signature scores separately for the 50 Hallmark pathways (51), then for the gene targets of BA9 or blood miRNAs and for WGCNA modules using summed z-scores. We then assessed the association using Pearson Correlation Coefficient, with significance achieved for *p* < 0.05. Correlation heatmaps were plotted using the Python language scientific library.

### 2.8. Statistical significance of overlap between brain and blood

Overlaps between lists of terms in brain and blood tissue (significant genes, pathways) were determined and represented using Venn diagrams. The statistical significance of the overlaps was determined using the DynaVenn approach (52).

## 3. Results

**FIGURE 1:**
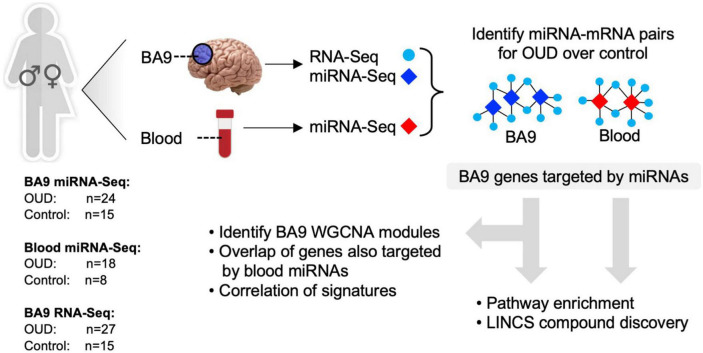
Analytical approach overview. RNA-sequencing was carried out in brain tissue collected postmortem from individuals with opioid use disorder (OUD) and controls. smallRNA-seq was carried out in both brain and blood tissue from a subset of the same individuals. Dysregulated miRNA–RNA networks were inferred, and miRNA target genes were further investigated using pathway enrichment, repurposable drug identification, and correlation-based network analysis.

To explore the potential role of miRNAs as regulators of transcription in opioid-exposed brains, we employed a multi-pronged sequencing and analytical approach focusing on the dorsolateral prefrontal cortex (DLPFC, Brodmann area 9, BA9) as this is a key cerebral area involved in inhibition and impulsivity, functions that are altered in substance use disorders (53). RNA-Sequencing (RNA-Seq) was carried out in bulk tissue from the DLPFC of individuals with OUD and controls, as we previously described (5). smallRNA-Seq, to identify miRNAs, was also performed in both BA9 and blood collected from the same donors. Dysregulated miRNA networks were inferred, and miRNA target genes were further examined using pathway enrichment, repurposable drug identification, and correlation-based network analysis. An overview of our analytical approach is outlined in Figure 1.

Demographic information of subjects is provided in Table 1, with additional detailed information previously described (5). The control group was 87% male with an average age of 55 years, and the OUD group was 56% male with an average age of 39 years. Controls were statistically more likely to be male than OUD (*p* < 0.05, Fisher’s Exact test), and statistically older than OUD (two-tailed Student’s *t*-test, *p* < 0.001, 95% CI of difference [7.31–24.69]).

**TABLE 1:** Demographic information of samples used for RNA-sequencing and smallRNA-sequencing.

Demographics	Control	OUD
Total number of samples	15	27
Males (%)	13 (87%)	15 (56%)
Age mean (SD)	55 (14)	39 (13)
PMI mean in hours (SD)	29 (7)	26 (9)
Ethnicity (White/Black/Hispanic/Asian)	8/4/2/1	25/5/0/0

OUD, opioid use disorder; PMI, postmortem interval.

### 3.1. miRNA–mRNA networks are dysregulated in OUD

**FIGURE 2:**
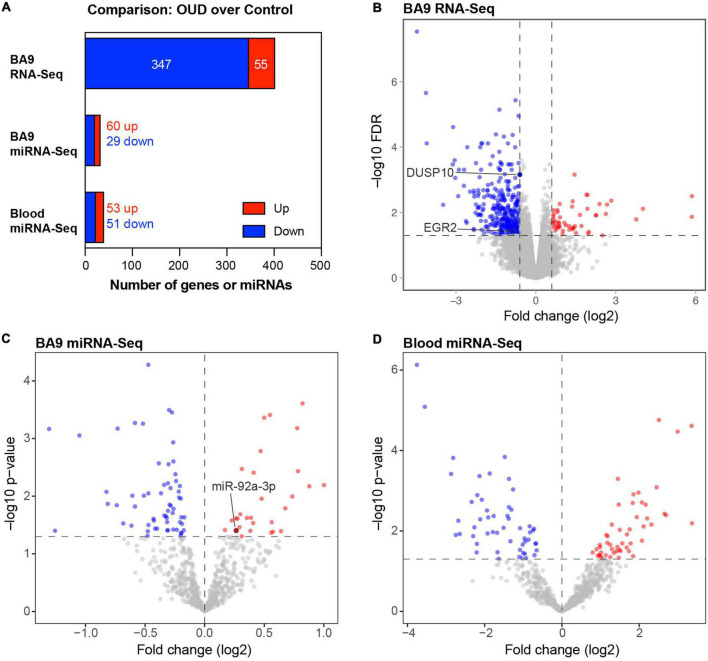
Differentially expressed protein-coding genes and microRNAs in brain and blood from individuals with opioid use disorder (OUD). **(A)** Summary of differentially expressed miRNA–mRNA network components. Volcano plots of BA9 protein coding genes **(B)**, BA9 miRNAs **(C)**, and blood miRNAs **(D)**, comparing OUD to controls. Genes highlighted in panel **(B)**, DUSP10 and EGR2, are targeted by miR-92a-3p, highlighted in panel **(C)**.

**FIGURE 3:**
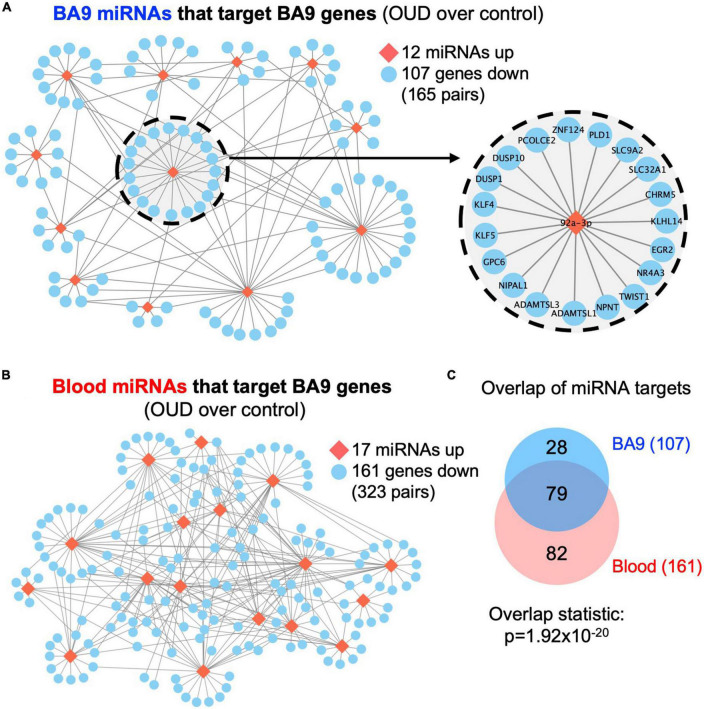
Dysregulated miRNA–mRNA networks in brains from opioid use disorder (OUD) donors. Using the significant dysregulated protein coding genes measured in BA9, we determined enriched miRNA targets based on the OUD-associated miRNA candidates measured in panel **(A)** BA9 and **(B)** blood via the mirDB compendium. **(C)** Overlap of down regulated genes targeted by miRNAs in BA9 and blood. Network of genes targeted by miR-92a-3p is enlarged in panel **(A)**.

We used RNA-seq to profile bulk BA9 tissue from 15 controls and 27 OUD cases. Employing stringent criteria of FDR-adjusted *p* < 0.05 and fold change exceeding 1.5x, we determined differential expression of 402 protein coding genes, with 55 induced in OUD and 347 decreased, after controlling for age, sex, PMI, RIN, and pH (Figure 2 and Supplementary Table 1A). We used a criterion of nominal unadjusted *p* < 0.05 to screen for miRNA network candidates based on differential expression of miRNAs, controlling for the same demographic and clinical covariates. From this analysis, we identified 89 differentially expressed miRNAs in BA9, with 29 induced in OUD and 60 suppressed (Figure 2C and Supplementary Table 1B). In blood, we identified 104 differentially expressed miRNAs, with 51 increased in OUD and 53 decreased (Figure 2D and Supplementary Table 1C). We then used the SigTerms bioinformatics platform (35, 36), developed by our group, to extract biologically significant miRNAs and their target mRNA pairs underlying OUD. This approach integrated our sequencing data with miRNA target prediction algorithms from mirDB (37) to identify miRNA–mRNA target pairs that are oppositely correlated in expression, applying a stringent statistical criterion with significance achieved at FDR-adjusted *p* < 0.05. Using the miRNA candidates identified in BA9 (Figures 2A, C), we identified 12 OUD up-regulated miRNAs that drove, at a stringent FDR-adjusted *p* < 0.05, a network of 107 OUD down-regulated genes (Figure 3A and Supplementary Table 2A). Using blood miRNA candidates (Figures 2A, D), we identified 17 OUD up-regulated miRNAs with targets enriched in 161 BA9 down-regulated genes (Figure 3B and Supplementary Table 2B). We did not identify overlap between potential BA9 and blood miRNA regulators. However, overlap of their target genes was robust, with 79 down-regulated genes enriched for targets of both BA9 and blood miRNAs (Figure 3C, overlap statistic *p* = 1.92 × 10^–20^). We then conducted Pearson Correlation Coefficient analysis between expression of the 29 miRNAs with significant mRNA targets in either BA9 or blood tissues. Twenty-three out of 29 miRNAs were commonly detected in both BA9 and blood samples (10 of the 23 in BA9 and 13 of 23 in blood) but only one of these, miR-340-5p, was significantly correlated between BA9 and blood (PCC = −0.46 and *p* = 0.02; Supplementary Figure 1C and Supplementary Table 2C). miR-340-5p was detected in blood samples and targeted 42 BA9 mRNAs (Supplementary Figure 1D and Supplementary Table 2B).

The direct (canonical) mode of miRNA-driven regulation of mRNA is that of transcription suppression, e.g., up-regulated miRNA targets are enriched in down-regulated genes and conversely down-regulated miRNA targets are enriched in up-regulated genes. However, indirect (non-canonical) regulation is possible via intermediate lncRNA modulation (54). As such, we also evaluated miRNA and coding gene changes in the same direction. Based on BA9 miRNA candidates, we identified 21 down-regulated miRNAs with targets enriched in 171 down-regulated genes (Supplementary Figure 1A and Supplementary Table 2A). Interestingly, the number of miRNA–mRNA pairs for non-canonical regulation exceeded the number for canonical regulation. Whereas a similar picture emerged using blood miRNA candidates, the difference between canonical and non-canonical miRNA–mRNA pairs was not as striking (Supplementary Figure 1B and Supplementary Table 2B). Specifically, 23 down-regulated miRNAs targeted 170 down-regulated genes.

### 3.2. Pathway enrichment and drug repurposing via targeting miRNA–mRNA networks

**FIGURE 4:**
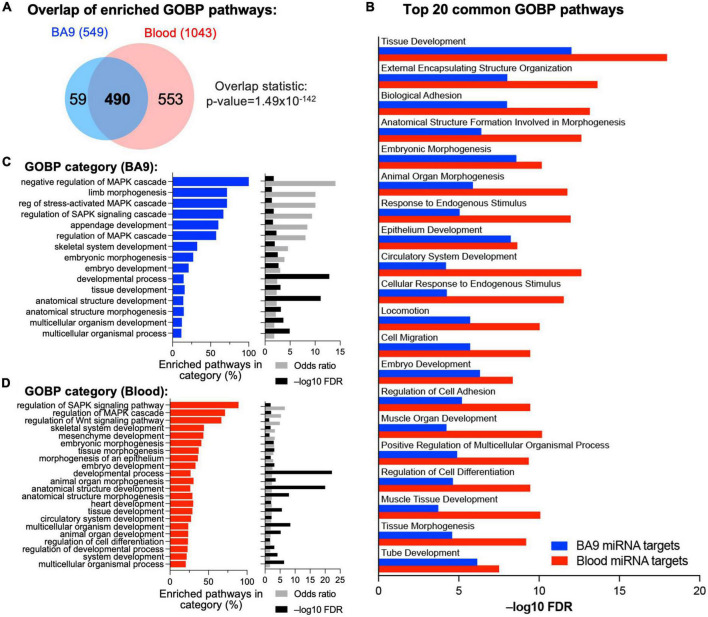
Integrative analysis of miRNA–mRNA networks. **(A)** Pathway enrichment was carried out with the enriched gene targets of BA9 and blood miRNAs using the Gene Ontology Biological Processes compendium, with overlap of enriched pathways shown as a Venn diagram. **(B)** Top 20 common GOBP pathways between BA9 and blood miRNA targets. **(C)** List of GO terms enriched in the 549 significant pathways in BA9 miRNA targets. **(D)** List of GO terms enriched in the 1,043 pathways significant in blood miRNA targets. For panels **(C,D)**, the percent of pathways associated with a GO term, odds-ratio, and –log10(FDR) are shown.

Based on BA9 miRNAs/BA9 gene networks and blood miRNAs/BA9 gene networks, we determined enriched Gene Ontology pathways using the MSigDB approach based on hypergeometric distribution, with significance achieved at FDR < 0.05 (Figure 4A and Supplementary Table 3A). Reassuringly, 490 pathways were enriched in gene targets of both BA9 miRNAs and blood miRNAs (Figure 4A, overlap statistic *p* = 1.49 × 10^–142^). Interestingly, targets of both BA9 miRNAs and blood miRNAs enriched for similar processes; whereas development and morphogenesis were the dominant dysregulated biological pathways and processes, another common term among the top 20 enriched pathways was cell adhesion (Figure 4B). Based on the observation that sometimes multiple related pathways differ only through a relatively small number of genes relative to the pathway size, the Revigo method has been developed to summarize enrichment or depletion at the level of Gene Ontology terms (55). Using this approach, the 549 GOBP pathways significant in the BA9 miRNA targets were enriched for developmental terms, as well as MAPK signaling and Stress Associated Protein Kinase signaling (SASPK) (Figure 4C and Supplementary Tables 3B, D). Similarly, the 1,043 pathways significant in the blood miRNA targets enriched both for developmental terms, and MAPK and SAPK signaling, further showing functional redundancy between the gene targets of BA9 and blood miRNAs (Figure 4D and Supplementary Tables 3C, D).

Repurposing of compounds based on gene signatures using the Library of Integrated Network-Based Cellular Signatures (LINCS) L1000 database (43) has generated effective compound candidates in numerous disease systems, including alcohol use disorder (56, 57). Smaller, refined gene signatures could be more effective to guide pharmacotherapy interventions via drug repurposing. Thus, we interrogated the LINCS database with our miRNA–mRNA network genes generated based on either BA9 miRNAs or blood miRNAs. Interestingly, BA9 and blood miRNA targets led to 173 common medications with an absolute score over 90 (Supplementary Figure 2A, overlap statistic *p* = 4.8 × 10^–48^; Supplementary Table 4) and 24 common compounds with an absolute score over 98, including dopamine receptor antagonist, JNK inhibitors, and CDK inhibitors (Supplementary Figure 2B).

### 3.3. WGCNA reveals miRNA–mRNA networks associated with individual cell types

**FIGURE 5:**
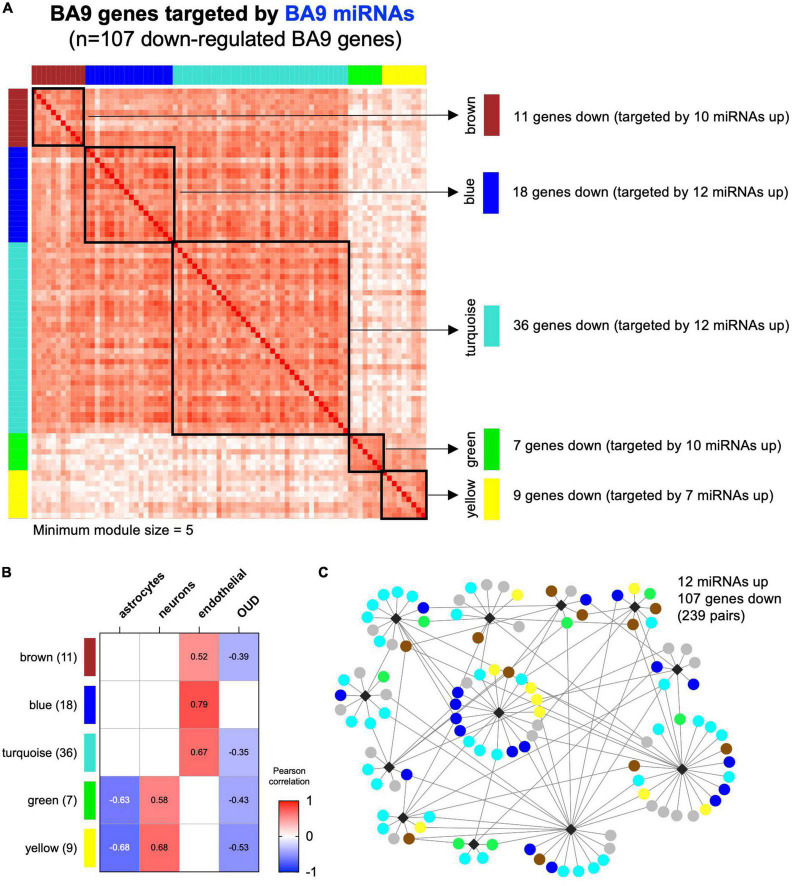
Correlation-based analysis of BA9 canonical miRNA–mRNA network genes. **(A)** Weighted gene correlation network analysis (WGCNA) was carried out for the 107 BA9 gene targets of BA9 miRNAs, identifying five distinct gene modules using a minimum module size of 5. The gray module of 26 uncorrelated genes was not included. **(B)** Association with brain cell type abundance (astrocytes, neurons, and endothelial cells) and OUD diagnosis was evaluated for each module. **(C)** Module membership is indicated in the network comprised of increased miRNAs and decreased genes, indicating the turquoise, blue, brown, green, and yellow WGCNA module genes.

Using CIBERSORT, we inferred cell type composition of the BA9 bulk tissue samples based on a collection of brain cell type signatures (42). Next, we employed WGCNA (40) to identify modules in the BA9 miRNA–mRNA network of 107 genes, identifying five modules ranging from 7 to 36 genes (Figure 5A). A sixth module, gray, contained 26 genes not clustered in any other module. By using the brain cell-type composition and the OUD status as traits, we performed module-trait association analysis, identifying WGCNA modules significantly associated with the relative proportion of astrocytes, neurons, or endothelial cells and also with OUD status (Figure 5B). The yellow and green modules associated negatively with astrocyte abundance and positively with neuron abundance (Figure 5B). The largest module, turquoise, as well as the brown and the blue modules associated positively with endothelial cell abundance. The turquoise, brown, green, and yellow modules associated negatively with OUD status and comprised the majority of the canonical miRNA–mRNA network driven by up-regulated miRNAs and down-regulated genes (Figures 3A, 5C).

As previously indicated, even though the network miRNAs inferred from BA9 and blood were non-overlapping, 79 down-regulated gene targets overlapped (Figure 3C and Supplementary Figure 3). Robust overlaps were also observed between the BA9 WGCNA gene modules and blood miRNA–mRNA network targets; the brown module was fully targeted by blood miRNAs (11/11 genes), the blue module showed 95% overlap (17/18 genes) and the turquoise module showed a 75% overlap (27/36 genes). The rest of the modules showed 56–57% overlap with blood miRNA gene targets (Supplementary Figure 3 and Supplementary Table 5).

**FIGURE 6:**
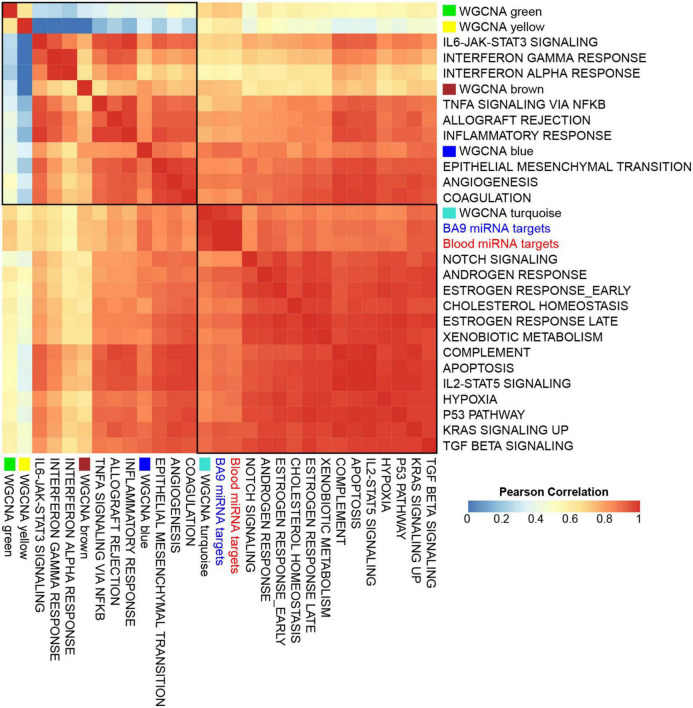
Gene set signatures for miRNA targets show robust and distinct correlation patterns. Gene signature scores were derived for opioid use disorder (OUD) down-regulated BA9 and blood miRNA targets, as well as for WGCNA modules, and comprehensive correlations were computed with the 50 Hallmark pathways over a collection of BA9 bulk tissue transcriptomes from 425 control brain samples from the GTEx project. Presented here is a subset of the Hallmark pathways, showing that OUD down-regulated targets of BA9 and blood miRNAs and the WGCNA turquoise module correlated with Hallmark pathways for Notch signaling, apoptosis, hypoxia, and TGFß signaling; WGCNA modules brown and blue correlated with immune system, inflammatory pathways, angiogenesis, and coagulation.

To assess if the WGCNA modules associated with distinct pathways, we first downloaded a database of 425 transcriptomes of control BA9 tissues compiled by the Genotype Tissue Expression (GTEx) project (50), then computed gene signature scores for genes targeted by either BA9 or blood miRNAs, as well as each of the WGCNA modules, and for a reference collection of the 50 Hallmark pathways (51). Strikingly, the signature scores for miRNA targets showed distinct correlation patterns across modules: the yellow and green modules correlated negatively, while brown and blue modules correlated positively, with immune system and inflammatory pathways, angiogenesis, and coagulation pathways. The turquoise module correlated positively with Notch signaling, apoptosis, hypoxia, and TGFß signaling (Figure 6 and Supplementary Figure 4).

## 4. Discussion

In this study, we performed an integrated analysis of RNA-seq and smallRNA-seq data from same-subject postmortem brain (BA9) and blood samples to explore the hypothesis that miRNAs and their mRNA targets are critical drivers of opioid-induced neurobiological alterations. This is the first study of its kind to investigate miRNA–mRNA networks for OUD in corresponding brain and blood from the same subjects. Overall, we identified robust miRNA–mRNA networks in both BA9 brain and blood. Although there was no overlap between differentially expressed miRNAs in BA9 and blood, we found strong overlap among the differentially expressed target genes of the network miRNAs from both tissues. In addition, the corresponding enriched pathways derived from miRNA–mRNA networks in brain and blood were highly overlapping, with tissue development, morphogenesis, and pathways related to circulatory system development among the dominant enriched biological processes. A GO term enrichment analysis further identified functional similarities in MAPK and Stress Associated Protein Kinase (SAPK) signaling. Further, using WGCNA, we identified cell-type specific miRNA targets, particularly in astrocytes, neurons, and endothelial cells, associated with OUD transcriptomic dysregulation. The largest module identified by WGCNA (turquoise - comprised of 36 genes) was positively associated with endothelial cells, and correlated with Hallmark pathways for Notch signaling, apoptosis, hypoxia, and TGFß signaling providing evidence for a role of endothelial cells in opioid-induced brain alterations, as we have previously reported (5).

Of interest, the identified miRNA targets included genes we previously found to be involved in endothelial cell function, cytokine signaling, and angiogenesis pathways associated with OUD, including *EGR1*, *EGR2*, *EGR4*, *NR4A2*, and *DUSP10* (5). Among the differentially expressed and enriched miRNAs was miR-92a-3p, which has been previously found to be dysregulated in blood from male subjects after hydromorphone or oxycodone treatment (58). Our bioinformatics analysis identified miR-92a-3p to be significantly up-regulated in BA9, and its targets *EGR2* and *DUSP10* (59, 60) to be significantly down-regulated. miR-92a is highly expressed in endothelial cells, which regulate vascular endothelial function. Overexpression of miR-92a in endothelial cells blocks angiogenesis and administration of a miR-92a inhibitor enhances recovery of damaged tissues in a mouse model of ischemia (61).

miR-92a acts through its targets via the p38 MAPK signaling pathway (62, 63), which we previously identified to be significantly enriched in OUD (5). Additional support for a role of MAPK pathways in OUD pathophysiology was found by our GO terms enrichment analyses in pathways identified by miRNA–mRNA networks in BA9 or blood.

Leveraging a collection of control brain transcriptomes from the GTEx project, we identified a correlation of OUD miRNA targets with hypoxia, TGFß, angiogenesis, coagulation, immune system, and inflammatory pathways. TGFß signaling can contribute to the pathogenesis of cardiovascular diseases (64), and *TGFB2* was among the top differentially expressed genes in our previous study of gene dysregulation in OUD postmortem brain (5).

Altogether, our current results using a multi-omics approach support previous findings in human and animal studies of neurovascular alterations as a consequence of opioid abuse (65–75), and establish miRNA–mRNA networks perturbed convergently in brain and blood of opioid users. Several peripheral blood miRNA candidates, including miR-369-3p (22 targets, including EGR1 and EGR2) and miR-627-3p (25 targets including EGR1, DUSP4, and DUSP10) should be investigated further as surrogate biomarkers for their target coding genes in brain. Importantly, the American Heart Association recently advised on the risk of opioid use for neurovascular complications (76), underscoring the urgency of research aimed toward understanding mechanisms underlying opioid-induced neurobiological alterations and treatments aimed at preventing them. Further preclinical studies in animal or cell models are needed to clearly define these mechanisms.

Limitations of this study include the small sample size, which precluded our ability to match subjects for age and sex. Additionally, although sex and age were controlled for in all analyses, controlling for these covariates limits the exploration of age and sex as important factors contributing to gene expression. Further, we used cell-type deconvolution of bulk RNA-seq data for determination of cell-specific effects. Future studies using single cell RNA sequencing technology are needed to validate the gene correlation findings in specific cell types. In summary, our findings suggest that miRNA-driven mRNA dysregulation in OUD is profound and potentially realized through redundant and systemic alternatives. Our study demonstrates the utility of analyzing miRNA networks to facilitate new avenues of mechanistic explorations that could lead to the development of novel therapeutic approaches to minimize or potentially reverse opioid-induced brain abnormalities.

## Data availability statement

The dataset used in this study (Bulk RNA-Seq) was previously published (5) and can be found at NCBI GEO (GSE182321). miRNA-Seq data generated for this study has been deposited at NCBI GEO (GSE221515; https://www.ncbi.nlm.nih.gov/geo/).

## Ethics statement

The studies involving human participants were reviewed and approved by University of Texas Health Science Center at Houston Institutional Review Board. Written informed consent for participation was not required for this study in accordance with the national legislation and the institutional requirements.

## Author contributions

CW-B and CC: conceptualization, resources, supervision, and funding acquisition. SG, EM, TG, RK, and CC: methodology, software, and formal analysis. EM, LS, TM, GF, SS, AT, TK, and PG: investigation and data curation. SG and CC: visualization. SG, EM, CC, and CW-B: writing – original draft of the manuscript. All authors were writing – review and editing and approved the submitted version.
