# Supplementary material for: MicroRNA–mRNA networks are dysregulated in opioid use disorder postmortem brain: Further evidence for opioid-induced neurovascular alterations
Source: Front Psychiatry. 2023 Jan 12;13:1025346. doi: 10.3389/fpsyt.2022.1025346 (PMC9878702; doi:10.3389/fpsyt.2022.1025346)
Supplement: Supplementary file 1 [file Data_Sheet_1.PDF]

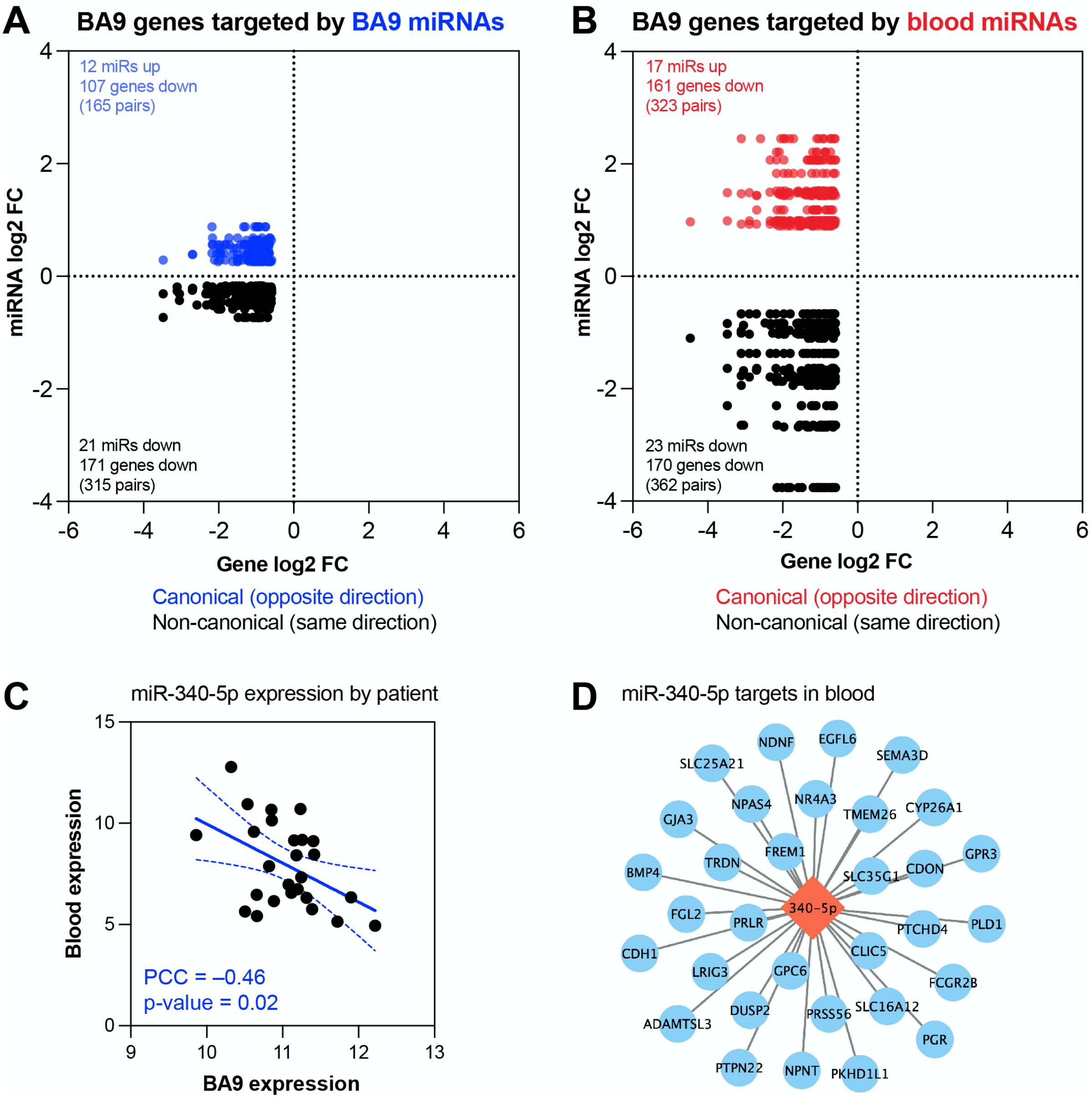

Supplemental Figure 1. Evaluation of canonical and non-canonical miRNA-mRNA networks using BA9 and blood microRNAs. Using differentially expressed mRNAs measured in BA9 at a stringent FDR<0.05, we assessed enrichment of miRNA targets based on differentially expressed miRNAs in (A) BA9 and (B) blood. We assessed canonical miRNA-mRNA regulation (e.g. opposite direction) and non-canonical miRNA-mRNA regulation (e.g. same direction). Some genes were enriched as miRNA targets under both canonical and non-canonical regulation. (C) Scatterplot showing the correlation of blood network miRNA miR-340-5p expression measured in log2(CPM) between BA9 and Blood tissue samples. (D) Enriched targets of Blood miRNA miR-340-5p in BA9 mRNAs significant at linear FC≥1.5 and FDR<0.05.

A **Overlap of potential therapeutic compounds (score ≥90):**

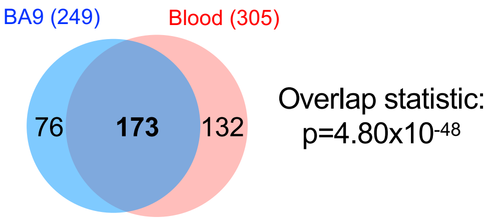

B

ID	Name	Description	BA9 compounds	Blood compounds
BRD-K69032158	diprotin-a	Dipeptidyl peptidase inhibitor	99.86	98.61
BRD-U82589721	HG-5-113-01	Protein kinase inhibitor	99.68	99.72
BRD-A31007383	SDZ-WAG-994	Adenosine receptor agonist	99.51	98.71
BRD-K16485616	mocetinostat	HDAC inhibitor	99.05	99.26
BRD-K08554278	bisbenzimide	DNA binding agent	98.97	98.17
BRD-K69600043	thiethylperazine	Dopamine receptor antagonist	98.80	99.33
BRD-K96402602	farnesylthiotriazole	PPMTase inhibitor	98.74	99.37
BRD-A04553218	chlorphenamine	Histamine receptor antagonist	98.59	99.72
BRD-K10467831	tibolone	Androgen receptor agonist	98.52	98.13
BRD-A00758722	noretynodrel	Progestogen hormone	98.34	99.09
BRD-K43389675	daunorubicin	RNA synthesis inhibitor	98.34	98.17
BRD-K11636097	JNJ-7706621	CDK inhibitor	98.27	98.27
BRD-U51951544	ZG-10	JNK inhibitor	98.24	98.10
BRD-A30437061	camptothecin	Topoisomerase inhibitor	98.20	98.34
BRD-K13566078	BMS-345541	IKK inhibitor	98.20	98.34
BRD-K11927976	ER-27319	Mediator release inhibitor	98.20	98.20
BRD-K19220233	JNK-9L	JNK inhibitor	98.20	98.20
BRD-A11702965	chromomycin-a3	DNA binding agent	98.20	98.09
BRD-K13390322	AT-7519	CDK inhibitor	98.17	98.13
BRD-K78126613	menadione	Mitochondrial DNA polymerase inhibitor	98.10	99.33
BRD-K99545815	PF-562271	Focal adhesion kinase inhibitor	98.10	98.03
BRD-K06426971	ryuvidine	Histone lysine methyltransferase inhibitor	98.06	99.61
BRD-K22878149	SB-205607	Delta 1 opioid receptor agonist	98.06	98.38
BRD-A15010982	HU-211	Glutamate receptor antagonist	98.03	98.73

Supplemental Figure 2. Overlap between repurposable medications predicted based on targets of BA9 and blood miRNAs. A. Repurposable compounds were ranked based on their anti-correlation with the gene targets detected in miRNA-mRNA networks from BA9 and blood. Overlap of compounds with an absolute score above 90 are shown. B. Common medications detected in both BA9 and Blood miRNA targets with an absolute score above 98.

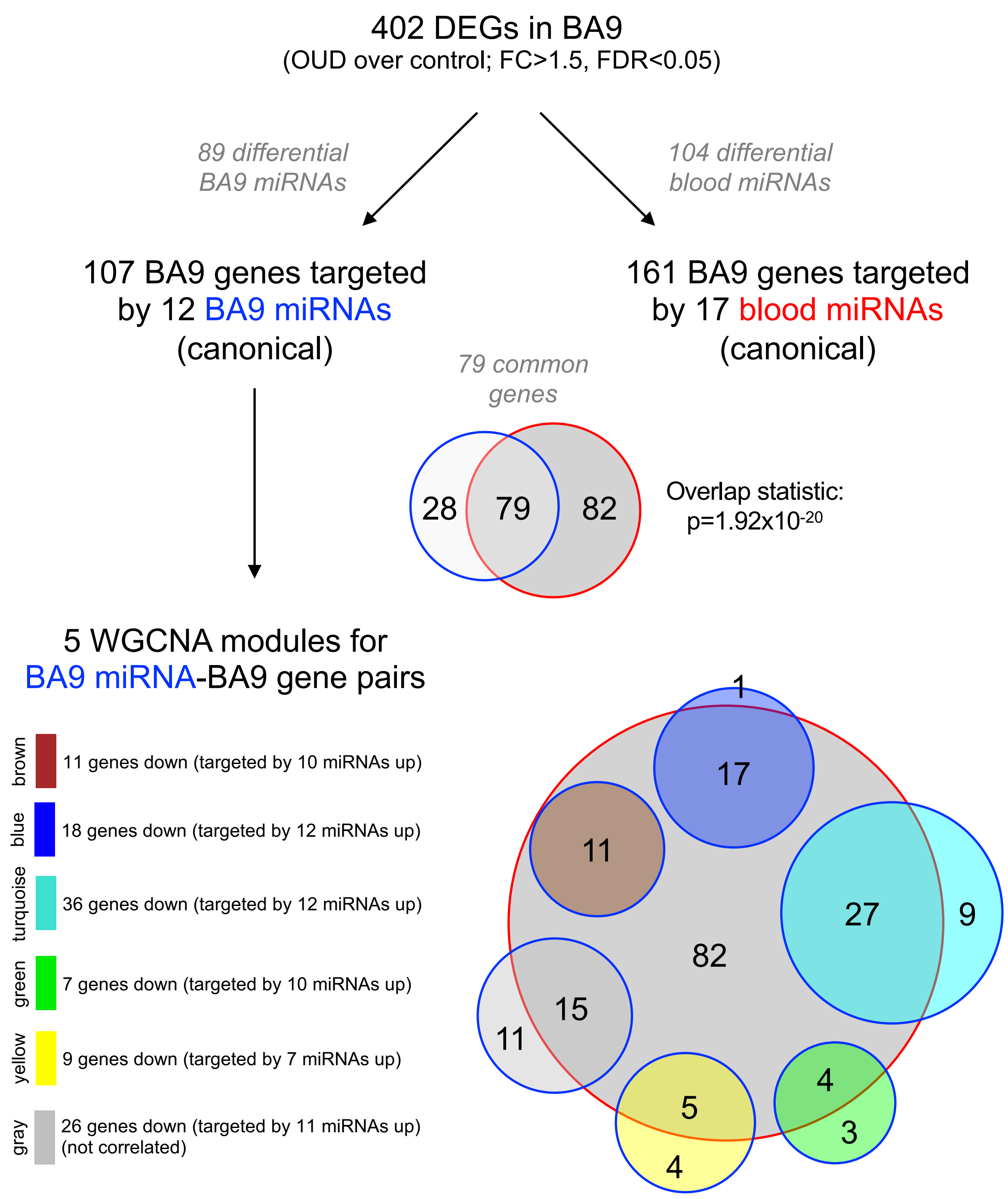

Supplemental Figure 3. Overlap between WGCNA modules and targets of blood miRNAs. Seventy-nine gene targets were common between BA9 miRNAs and blood miRNAs. WGCNA BA9 non-gray module overlap with blood miRNA targets ranges between 56% (yellow module) to 100% (brown module).

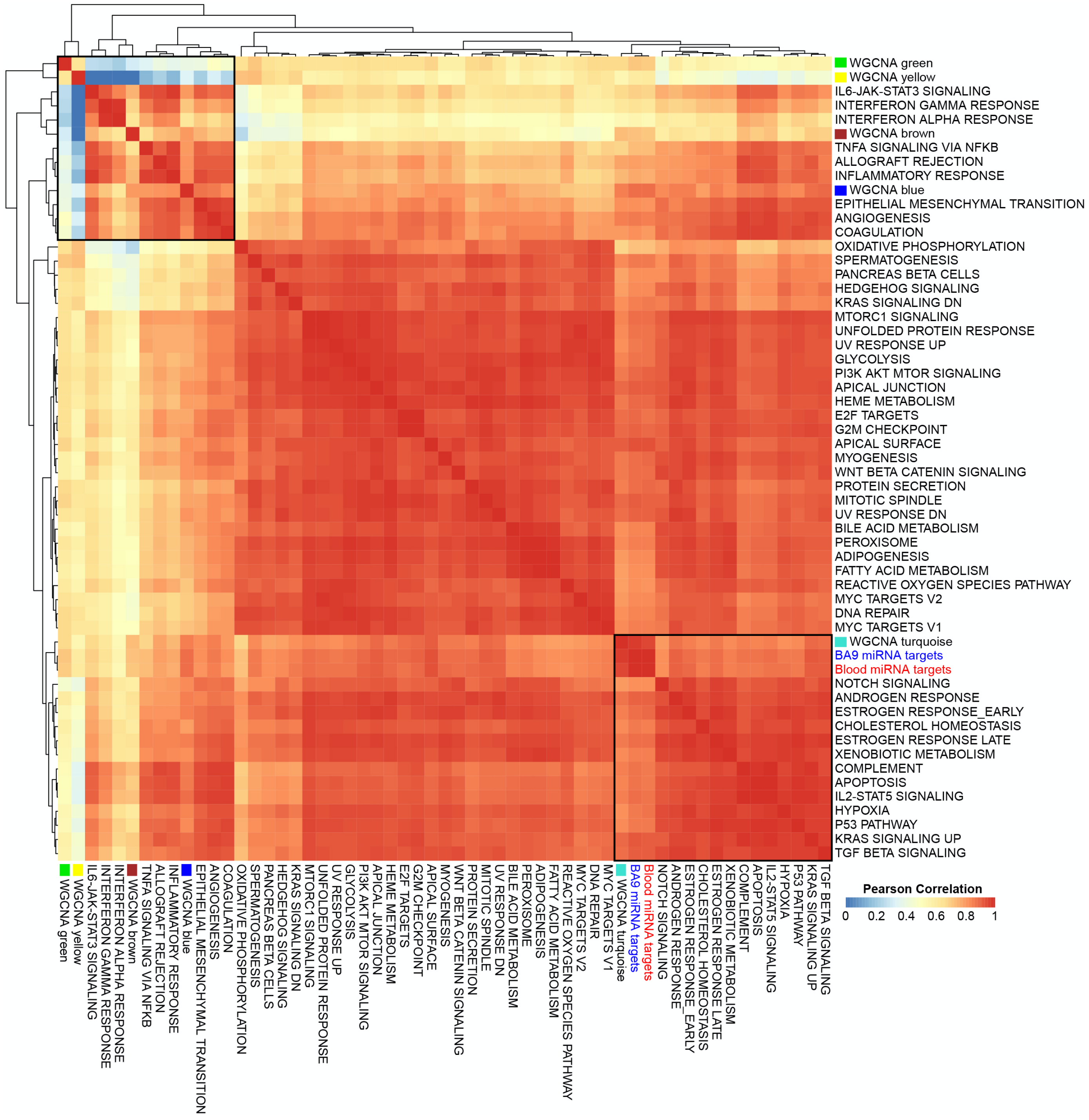

Supplemental Figure 4. Comprehensive correlation heatmap of gene set signatures for miRNA targets and Hallmark pathways. Gene signature scores were derived for OUD down-regulated BA9 and Blood miRNA targets, as well as for non-gray WGCNA modules, and comprehensive correlations were computed with the 50 Hallmark pathways over a collection of BA9 bulk tissue transcriptomes from 425 GTEx control brains.

Supplemental Tables

Supplemental Table 1. mRNA and miRNA network candidates. (A) Differentially expressed genes in OUD over Control tissue biopsies from BA9 brain at linear FC≥1.5 and FDR<0.05. (B) Screening of candidate network miRNAs from BA9 at p<0.05. (C) Screening of candidate network miRNAs from blood at p<0.05.

Supplemental Table 2. Genes targeted by miRNAs. miRNA-mRNA pairs as determined using miRDB in BA9 brain (A) or blood (B) for mRNAs significant at linear FC≥1.5 and FDR<0.05. (C) Correlation of log2(CPM) expression between BA9 and blood for 23 miRNAs with targets enriched in BA9 mRNAs significant at linear FC≥1.5 and FDR<0.05.

Supplemental Table 3. Pathway enrichment using genes targeted by miRNAs. (A) Enriched Gene Ontology Biological Process (GOBP) pathways by Over-Representation Analysis (ORA) using genes targeted by either BA9 or blood miRNAs. Significance (–log10 of the FDR), number of genes enriched for each pathway, and differentially expressed genes in each pathway are listed. (B) GO Terms for enriched Gene Ontology Biological Process (GOBP) pathways in BA9 miRNA targets including the pathways comprising them. (C) GO Terms for enriched Gene Ontology Biological Process (GOBP) pathways in Blood miRNA targets including the pathways comprising them. (D) Top GO Terms for enriched Gene Ontology Biological Process (GOBP) pathways in BA9 and Blood with comprehensive statistics.

Supplemental Table 4: Compounds identified using LINCS for BA9 genes targeted by BA9 or Blood miRNAs. For each compound we present the LINCS ID, name, description, and absolute scores in the BA9 miRNA targets and Blood miRNA targets.

Supplemental Table 5. WGCNA modules based on BA9 miRNA-mRNA network genes. Membership of differentially expressed genes for each module is indicated. Overlap with blood miRNA-BA9 mRNA network genes, in either a canonical or non-canonical fashion, are annotated.
